# Leakage-Free Benchmarking of Electronic Noses for Beef Freshness: A Signal-Richness Criterion for Model Selection

**DOI:** 10.3390/foods15162798

**Published:** 2026-08-10

**Authors:** Erkan Caner Ozkat

**Affiliations:** Department of Mechanical Engineering, Faculty of Engineering & Architecture, Recep Tayyip Erdogan University, Rize 53100, Türkiye; erkancaner.ozkat@erdogan.edu.tr

**Keywords:** electronic nose, beef freshness, machine learning, label leakage, food quality control

## Abstract

Low-cost metal-oxide-semiconductor (MOS) electronic noses promise rapid, non-destructive meat freshness screening, and published classifiers frequently approach perfect accuracy. Such figures are rarely tested against the two conditions that most inflate them: a target-derived label among the inputs, and random splitting of the correlated samples. Beef freshness is benchmarked here on a public 11-sensor, 12-cut MOS dataset using leakage-free leave-one-cut-out cross-validation in order to predict freshness class and total viable count (TVC) with paired significance tests. A gradient-boosted-tree pipeline is the strongest model (accuracy 0.81±0.10, macro-F1 0.68±0.15, TVC $R^{2}=0.77$), significantly outperforming a multi-scale attention convolutional network (macro-F1 0.50±0.15; p<0.001). The advantage of this study lies in the representation, not the model family: a network given the same window summaries reaches 0.64±0.17, indistinguishable from the tree. Near-perfect accuracy returns only when TVC is supplied as a feature or samples are split at random (macro-F1 0.97). Under nested, per-fold selection, a five-sensor subset matches the full array. On a rich BME688 heater profile dataset, the network surpasses the tree, an advantage that vanishes as the profile shortens to one step. Evaluation and representation, not architecture, govern reported performance; a signal-richness criterion predicts when a deep temporal model is justified.

## 1. Introduction

Meat spoilage is a food-quality and economic problem. Aerobic microbial growth degrades the proteins and lipids of the meat. It releases a characteristic mixture of volatile organic compounds (VOCs). These include biogenic amines, sulfur compounds, alcohols and short-chain aldehydes. Their headspace concentration rises as the microbial population grows. Aerobic spoilage of chilled beef is dominated by *Pseudomonas* spp. and Enterobacteriaceae [1,2]. Their proteolytic activity releases ammonia and the biogenic amines cadaverine and putrescine [3]. Sulfur volatiles such as hydrogen sulfide appear later and at lower headspace concentration under aerobic storage. Alcohols, aldehydes, and ketones accumulate throughout lipid oxidation and carbohydrate catabolism. The reference methods, aerobic plate counts, and chromatographic analysis of the volatiles, are accurate but slow, destructive, and laboratory-bound, and are therefore ill-suited to on-site screening. An electronic nose (e-nose) built from an array of low-cost metal-oxide-semiconductor (MOS) gas sensors offers a portable alternative. The array transduces the evolving VOC mixture into a multichannel electrical signal. From this signal, a model reads out a freshness grade and the underlying microbial load. That load is the total viable count (TVC) in $\log_{10}$ CFU $g^{-1}$. TVC indexes freshness and remaining shelf life rather than microbiological safety [2]. Pathogens can be present without a correspondingly elevated aerobic count, so an instrument of this kind screens quality, not safety.

Machine learning is the natural readout layer: a partially selective array’s response is high-dimensional and cannot be reduced to fixed thresholds. Food quality control has shifted the same way. Wang et al. [4] built an attention-augmented deep detector for the non-destructive inspection of tea. Liang et al. [5] benchmarked an attention-based one-dimensional convolutional network against classical learners for the hyperspectral traceability of fruit. Broader reviews report the same trend across electrochemical and gas sensor food-safety platforms [6,7,8,9]. For beef, e-nose systems paired with machine learning are already established, from IoT-enabled monitoring of beef quality [10] to MOS-based detection of meat adulteration [11]. Several report freshness-grade classifiers reaching accuracies close to 100%. Feyzioglu and Taspinar [12], using the same public corpus adopted here, report 100% accuracy with three ANOVA-selected features. Surjith et al. [13], again on this corpus, report 99.8% for the fresh-versus-spoiled task with a multi-modal RF–CNN–GRU ensemble. Wijaya et al. [14] reached comparable accuracy with a Random-Forest and AdaBoost ensemble and filter-based sensor selection. These figures are encouraging, but they are seldom tested against the two conditions that most easily produce them. First, the freshness grade is defined by thresholding TVC. Including TVC, or any near-deterministic function of it, among the inputs then leaks the answer into the model. Second, consecutive readings from one physical sample may be split at random between training and test. The model is then scored on near-duplicates of what it has already seen. Both conditions inflate performance in a way a deployed device would not. Against this background stands the denoising long–short-term-memory model of Wijaya et al. [15], which used grouped evaluation. It reports a more modest 94.83% four-class accuracy, treated here as the realistic reference bar.

This paper measures what a low-cost MOS-array beef e-nose achieves and what model class it warrants under a deliberately conservative evaluation. The contributions are six-fold. First, a leakage-free, significance-tested benchmark is provided. On a public 11-sensor, 12-cut beef dataset, both freshness grade and TVC are predicted using group-structured leave-one-cut-out (LOCO) cross-validation. A paired test shows that a gradient-boosted-tree pipeline significantly outperforms a multi-scale attention convolutional network. A channel-importance analysis and a deployment-cost measurement are also reported. Second, a controlled account of the inflation is given. On the same data, near-perfect accuracies are reproduced by the two conditions above, each isolated as a single-variable experiment. Third, a signal-richness criterion is formulated and tested. In earlier work on tea grading, Tasdemir et al. [16] found that grade information lies in the temporal shape of VOC release. Controlled heating reveals this shape, which summary statistics discard. The beef signal, sampled once per minute, has no such intra-cycle shape. A confound-controlled dose-response on a rich BME688 heater profile dataset [17] confirms that a deep temporal model only helps when the measurement carries waveform structure. Fourth, the advantage of the classical pipeline is decomposed into representation and model family, and is shown to rest on the representation. Fifth, sensor selection is nested inside each training fold, which gives an unbiased estimate of what a reduced array costs. Sixth, the between-device distribution shift is quantified and calibration-transfer strategies are evaluated. What is not new should also be stated plainly: the datasets, the sensor array, and the model families are all drawn from published work. The contribution is the evaluation methodology, the model selection criterion, and the corrected picture of achievable performance that follows from them.

The remainder of the paper is organised as follows. Section 2 reviews the related literature. Section 3 describes the dataset, signal processing pipeline, proposed architecture, and training protocol. Section 4 presents the results. Section 5 discusses the implications and limitations. Section 6 concludes.

## 2. Related Works

Artificial intelligence has become the dominant methodology for food safety analysis and quality control, and recent work shows prevailing patterns. Wang et al. [4] drew on primate visual mechanisms to build an improved YOLOv13 detector with sparse self-attention. It targets the non-destructive detection of foreign matter in Pu-erh tea. Liang et al. [5] proposed a one-dimensional convolutional network with an efficient channel-attention mechanism for the hyperspectral origin identification of fragrant pears. They benchmarked it against linear discriminant analysis, random forests, *k*-nearest neighbours, and support-vector machines. Chen et al. [18] assessed beef freshness from visible and near-infrared spectra with interpretable machine learning. The review of Hussain et al. [9] surveys how these methods are reshaping the food industry. Reviews of AI-assisted chemical and electrochemical sensing for food and environmental monitoring report a common organising theme. Learning-based models improve sensitivity, feature selection, and robustness across these platforms [6,7,19,20,21,22,23,24]. The survey of Yu et al. [8] identifies classification, feature selection, and drift handling as the central problems for machine learning-assisted gas sensor arrays.

The MOS gas sensor array read as an e-nose is closest to the present task and has been applied across meats. Damdam et al. [10] built an IoT-enabled e-nose that monitors beef quality and flags spoilage in real time. Huang and Gu [11] used an MOS-based e-nose with machine learning to quantify meat adulteration. Han et al. [25] detected beef adulterated with pork using a low-cost colorimetric-sensor e-nose. Chen et al. [26] evaluated the quality and authenticity of Beijing-You chicken with an e-nose. This is a poultry analogue of the beef problem addressed in this work. Mohareb et al. [27] assessed beef-fillet freshness from a quartz-crystal-microbalance e-nose. Support vector machine ensembles classified sensory state and regressed microbial counts, with TVC among them. This is a classical model precedent for the joint class and TVC task pursued here. Beyond red meat, Feng et al. [28] coupled an IoT e-nose to spoilage detection for salmon during cold storage. Wang et al. [29] evaluated and dynamically predicted oyster freshness from a ten-sensor array. That work was cross-checked against gas chromatography-mass spectrometry and microbial counts. Two threads bear directly on the present contributions. Kong et al. [30] reduced a ten-sensor array to five or six sensors while holding accuracy above 95%. They cut redundancy by more than 40%, echoing the channel-importance result reported here. Enériz et al. [31] implemented sensor-fusion machine learning for beef classification and microbial-load prediction on a single field-programmable gate array—an edge-deployment precedent. MOS e-noses have also been used for fine-grained food classification and authentication [32], as well as chemo-resistive VOC arrays for breath diagnostics [33]. The MOS-array-plus-learning combination studied here was recently demonstrated by Tasdemir et al. [16] for black-tea grading. There, a single programmable sensor operated as a thermal-desorption profiler, and a raw-waveform deep model outscored feature-based classifiers. The study is the methodological predecessor of the present work.

Meat freshness sensing has also advanced along sensor front-ends that target specific spoilage markers. Li et al. [34] developed a PANI/silver-nanowire gas sensor for pork freshness. Yu et al. [35] built a silver-nanoparticle sensor array for meat freshness inspection, and Sun et al. [36] built a ratiometric fluorescent sensor for beef. Fengou et al. [37] detected meat adulteration with spectroscopy-based sensors. Optical and colorimetric biosensors follow the same logic in a single-marker form. Abbasi-Moayed et al. [38] discriminate biogenic amines through nanoparticle aggregation. Calabretta et al. [39] report a genipin colorimetric paper for biogenic amines in chicken. Li et al. [40] track volatile basic nitrogen in pork with a fluorescent metal–organic framework film. Calabrese et al. [41] use an odorant-binding-protein impedimetric biosensor for spoilage VOCs. Ham et al. [42] print a hydrogel that changes colour with ammonia. Pan et al. [43] read pH-sensitive pitaya-peel films with a smartphone and a multimodal deep model for fish freshness—an optical analogue of the readout problem addressed here. These devices are sensitive and inexpensive. Each reports one or a few markers at a single time point. That is not the evolving temporal response of a semiconductor array that the present models read.

The deep learning components compared against here are established for one-dimensional sensor signals. Altuwaijri and Muhammad [44] showed that a multi-branch convolutional network with several kernel sizes extracts multi-scale temporal features from raw signals. It does so more effectively than a single-scale network. This motivates the multi-branch stem of the deep comparator used here. Gao et al. [45] placed a one-dimensional convolutional network alongside PCA-LDA, random forests, and support vector machines—the classical-versus-deep design adopted here. Park et al. [46] argued for documented evaluation procedures and cross-batch testing in place of random splitting. That is the concern at the centre of this paper. Transfer across devices has been tackled with domain-adversarial learning. Wang and Gu [47] aligned e-nose feature distributions for cross-domain tea classification. That approach is revisited here for the present cross-dataset gap. For food freshness specifically, Hamidy et al. [48] predicted fish freshness from multi-angle images. Lu et al. [49] fused deep features with malondialdehyde and sulfhydryl measurements to predict oyster freshness. Denoising front-ends [50], one-shot CNN regression [51], and deep models for microbial detection [52,53] round out the toolkit. Deep learning on one-dimensional signals is also established in the present author’s earlier work. It spans laser-weld acoustic and photodiode monitoring [54,55] and vibration-based UAV diagnostics [56]. It also covers attention-based architectures on time series [57] and the benchmarking of classical learners across domains [58,59]. A recurring finding, encountered again here, is that a compact model can match or beat a larger deep network on a small dataset [54]. Machine learning and molecular modelling are also widespread across the life sciences. Examples range from SARS-CoV-2 inhibitor discovery [60] to the optimisation of in vitro plant regeneration [61]. In silico screening, similarly, guides drug design, with docking and molecular dynamics studies on BRPF1 [62] and GSK3β [63] inhibitors, as well as on anticancer bischalcones [64].

The specific beef corpus used here has an active methodological literature whose reported accuracies span a wide range. They run from the 94.83% of the grouped evaluation DWTLSTM [15] to near-perfect figures under feature reduction [12], ensembling [14], and multi-modal fusion [13]. The released corpus and its peer-reviewed descriptor make this the natural testbed [65,66]. Part of that spread is explained by evaluation choices rather than the models. The gap addressed here is therefore not the absence of models; it is the absence of a leakage-free, significance-tested benchmark, with a criterion for when a deep temporal model is warranted.

## 3. Materials and Methods

### 3.1. Primary Dataset: Beef, 11-Sensor MOS Array

The primary dataset is the public “various beef cuts” e-nose corpus deposited on Harvard Dataverse [66]. It comprises 12 independent beef cuts, each monitored for 2220 min at one reading per minute. This gives 26,640 time-stamped rows with no missing values. Each row records 11 MOS gas sensor responses (MQ135, MQ136, MQ137, MQ138, MQ2, MQ3, MQ4, MQ5, MQ6, MQ8, MQ9). It also records the measured TVC (in $\log_{10}$ CFU $g^{-1}$) and a four-level freshness label (excellent, good, acceptable, spoiled). The label is obtained by thresholding TVC, which is central to the leakage analysis below. Because the class is a deterministic function of TVC, any feature that reproduces TVC also reproduces the class. The classes are cleanly ordered in TVC (excellent 1.88–2.95 < good 3.03–4.00 < acceptable 4.03–4.94 < spoiled 5.00–5.76). The grade is a pure function of TVC: none of the 110 distinct TVC values in the corpus maps to more than one grade. The sharp split at TVC = 5.0 explains why models given TVC as an input trivially reach near-perfect accuracy. The label distribution is heavily imbalanced, with spoiled accounting for 59% of rows, so a majority-class predictor already reaches 0.595 accuracy. Macro-averaged F1 is reported throughout, and class weighting is applied as described in Section 3.3. Because each cut is one physical sample measured repeatedly, the 12 cuts define the grouping unit for cross-validation.

One structural property of the deposited corpus must be stated because it bounds what the benchmark can claim. The TVC series is not independent across cuts. Ten of the twelve sheets carry a partially identical TVC column; only Inside-Outside and Fat differ. TVC is recorded once per hour and held constant between updates, giving 37 distinct values over 2220 rows. Every cut crosses the spoilage threshold of 5.0 $\log_{10}$ CFU $g^{-1}$ at minute 901, and the grade boundaries fall at minutes 301/661/901 for ten cuts, 301/541/901 for Inside-Outside and 241/541/901 for Fat. The between-cut variance of the growth rate is accordingly $5.5\times 10^{-6}$, with mean rate 0.0973±0.0023 $\log_{10}$ CFU $g^{-1}$ $h^{-1}$ and a coefficient of variation of 2.4%. This is a property of the deposited reference series rather than a biological result, so inter-cut variability in spoilage rate cannot be estimated from this corpus. The label is, to within the three boundary shifts noted above, a deterministic function of elapsed time. The sensor signals are nevertheless acquired per cut, and leave-one-cut-out remains the correct protocol because it holds out an entire physical acquisition. The consequence for the present argument is direct. When the label is nearly a function of time, any protocol that lets adjacent minutes straddle the training-test boundary measures the ability to infer elapsed time rather than freshness.

**Figure 1:**
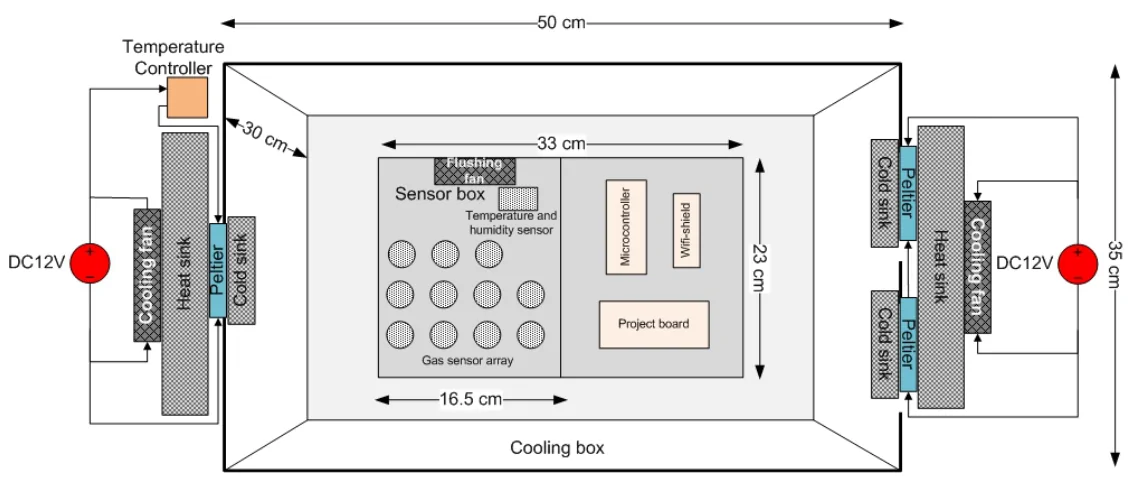
ENVBC acquisition system: a beef cut in the sampling chamber, read once per minute by the eleven-sensor MOS array for 2220 min [66].

**Figure 2:**
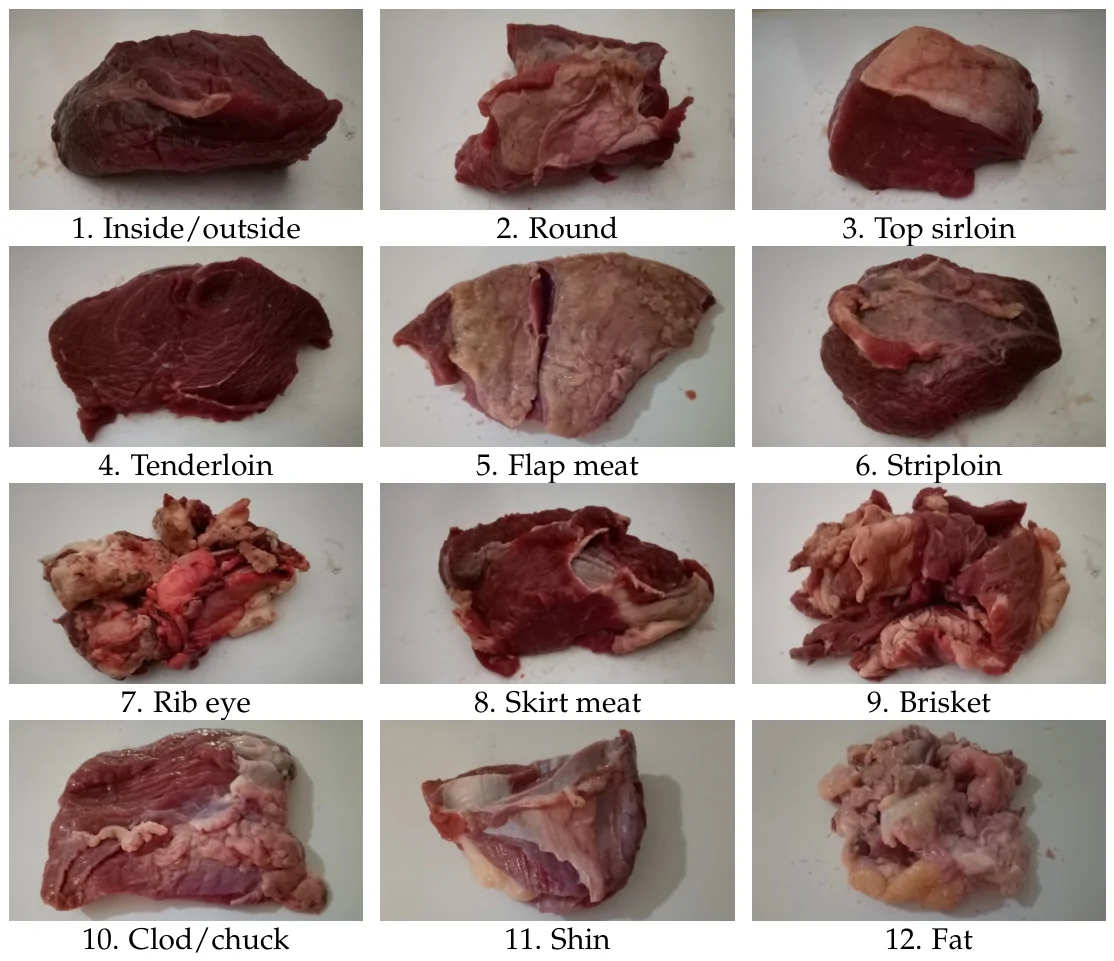
The twelve beef cuts of the ENVBC dataset, in table order [66].

Figure 1 shows the acquisition system, and Figure 2 shows the twelve cuts. Following the original protocol [12,66], each cut was placed in a sampling chamber. It was read once per minute using an Arduino-driven array of eleven MQ-series MOS sensors. Their targets span the volatiles of spoilage: ammonia (MQ137), hydrogen sulphide (MQ136), and alcohols, aldehydes and ketones (MQ138, MQ3). A range of combustible gases is covered by MQ2, MQ4, MQ5, MQ6, MQ8 and MQ9. MQ135 is additionally sensitive to nitrogen oxides, carbon dioxide, and benzene.

Table 1 maps each channel to its nominal target analytes and to the classes of spoilage volatile it reports. These devices are only partially selective, so a response is a weighted mixture over classes rather than a measurement of one compound [67,68]. This is why a learned readout layer is required and why the channel importance of Section 4.4 is read as an ordering over overlapping sensitivities rather than as chemical speciation.

### 3.2. Cross-Dataset Set and Rich-Signal Foil

For a cross-dataset robustness check, a second, independently acquired beef e-nose corpus by the same group is used, the “uncontrolled ambient conditions” set [65,66]. It holds five time-series from five separate beef cuts, 10,800 rows. The sensor array differs: nine MOS sensors plus in-chamber humidity and temperature, but without the MQ137 and MQ138 of the primary array. The two sets differ in physical samples and sensor complements. A primary-trained model is applied over the nine shared channels (MQ135, MQ136, MQ2, MQ3, MQ4, MQ5, MQ6, MQ8, MQ9). This is a genuine test of transfer across independent acquisitions. To isolate why a deep model does or does not help, a further substrate is used. In it, each measurement is a short waveform rather than a single reading. These are the BME688 (Bosch Sensortec GmbH, Reutlingen, Germany) heater profile datasets released with a portable odour-recognition study [17]. For every trace, they record the gas sensor resistance across a 10-step heater profile. CoffeePow-4 (3583 traces, four classes) and Aroma-7 (4750 traces, seven classes) are used. These substrates are different food matrices used only as a signal-richness contrast; the food-quality contribution of this paper rests on the beef/TVC result.

### 3.3. Leakage-Free Evaluation Protocol

Consecutive minutes within one beef cut are near-duplicates. A random split of rows then places near-identical samples in both training and test sets. Such a split measures memorisation rather than generalisation [69]. Leave-one-cut-out (LOCO) cross-validation is therefore used. In each of the 12 folds, all rows from one cut form the test set. The remaining eleven cuts form the training set. No row from a test cut is ever seen during training. The freshness class is never used as an input, and neither is TVC: the classification models see gas sensor signals only. Classification is scored by accuracy, macro-averaged F1, balanced accuracy, the Matthews correlation coefficient (MCC), and quadratic-weighted Cohen’s κ. The weighted κ credits the ordinal structure of the four grades. TVC regression is scored by RMSE and by a pooled $R^{2}$ over the concatenated out-of-fold predictions. The pooled $R^{2}$ is more stable under LOCO than a per-fold average. Class weights inversely proportional to class frequency are applied to the neural models. For the gradient-boosted trees, the weighted and unweighted fits are equivalent under this protocol (macro-F1 0.682 versus 0.684; paired p=0.966), and the unweighted fit is reported. Rebalancing uses weighting rather than synthetic oversampling. Interpolation schemes such as SMOTE would fabricate windows between autocorrelated, cut-grouped samples. That would produce physically implausible volatile trajectories and blur the very group boundaries that leave-one-cut-out is designed to protect. Every head-to-head comparison is tested with a paired Wilcoxon signed-rank test across the aligned folds. It is reported with the median paired difference and its bootstrap 95% confidence interval. The within-cut correlation that motivates this protocol can be quantified. Averaged over sensors and cuts, the autocorrelation of the raw channels is 0.959 at a lag of one minute, 0.932 at the eight-minute stride, and 0.877 at the sixty-minute window length. The integrated autocorrelation time is about 751 min, so a cut of 2220 readings carries roughly three independent observations and the full corpus carries roughly 36. Holding out one cut therefore removes about one-twelfth of the independent information, which is not an unreasonable share. A random split over rows, by contrast, places near-duplicates on both sides of the boundary.

### 3.4. Models

The two compared models use identical windows and folds. Sixty-minute windows are extracted per cut at an eight-minute stride. The sixty-minute length averages over short-term sensor and humidity fluctuations while still yielding a few hundred windows per cut. The stride sets how many overlapping windows each cut contributes. The overlap is only ever within a cut, and leave-one-cut-out holds out entire cuts. No test-cut window therefore shares a sample with a training window. The stride thus sets training-set size without leaking across the evaluation boundary. The gradient-boosted-tree pipeline represents each window by per-sensor summaries: mean, standard deviation, final value, range, and slope. This gives a compact, position-agnostic feature vector. Gradient-boosted trees are then used for classification and TVC regression.

**Figure 3:**
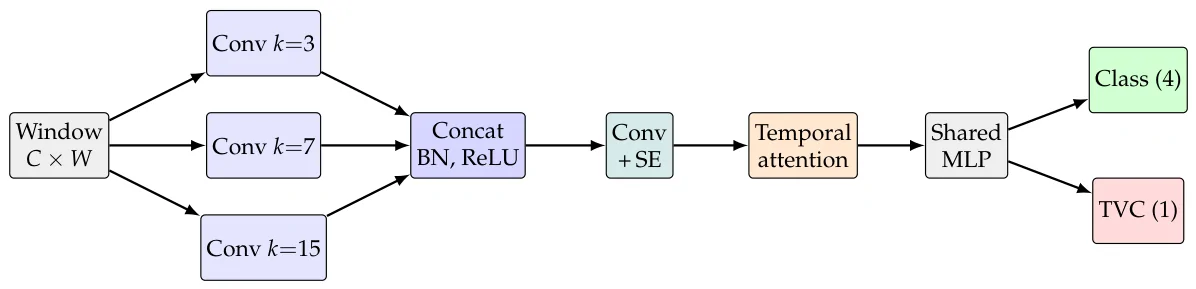
MS-CNN-Attention architecture (Equations (1)–(9)): multi-branch convolutional stem (kernels {3,7,15}), squeeze-and-excitation, temporal self-attention, and a shared representation feeding four-class and TVC heads.

The deep comparator is the multi-scale attention convolutional network (MS-CNN-Attention), summarised in Figure 3. It was introduced by the present author and colleagues for tea grading [16]. Let a window be $x\in R^{C\times W}$ with *C* sensor channels and *W* time steps. A multi-branch convolutional stem applies parallel one-dimensional convolutions with kernel sizes {3,7,15}. These are concatenated along the channel axis (denoted ∥) so that short and long temporal patterns are captured jointly:

(1) $$H=\mathrm{ReLU}\;\left(\mathrm{BN}\;\left({\mathrm{Conv}}_{3}\left(x\right)\;∥\;{\mathrm{Conv}}_{7}\left(x\right)\;∥\;{\mathrm{Conv}}_{15}\left(x\right)\right)\right)$$

with $H\in R^{C^{\prime }\times W}$ and $C^{\prime }=3b$ for branch width *b*. A refinement convolution then feeds a squeeze-and-excitation (SE) block

(2) $$H^{\prime }=\mathrm{ReLU}\;\left(\mathrm{BN}\;\left({\mathrm{Conv}}_{3}\left(H\right)\right)\right)$$

which reweights channels by their global response

(3) $$s=\sigma \;\left(W_{2}\;\mathrm{ReLU}\;\left(W_{1}\;{\bar{H}}^{\prime }\right)\right)$$

where ${\bar{H}}^{\prime }=\frac{1}{W}\sum_{t}H_{:,t}^{\prime }$ is the channel-wise temporal average and σ the sigmoid, giving the recalibrated feature map

(4) $$\tilde{H}=H^{\prime }⊙s$$

with ⊙ a per-channel scaling. A temporal self-attention layer assigns each time step *t* a weight

(5) $$\alpha_{t}=\frac{\exp\;\left(w_{a}^{⊤}{\tilde{H}}_{:,t}\right)}{\sum_{\tau }\exp\;\left(w_{a}^{⊤}{\tilde{H}}_{:,\tau }\right)}$$

and pools the sequence into a single context vector

(6) $$c=\sum_{t}\alpha_{t}\;{\tilde{H}}_{:,t}$$

A shared multilayer perceptron maps the context to a representation z=MLP(c), which feeds a four-class softmax classifier

(7) $${\hat{y}}_{\mathrm{cls}}=\mathrm{softmax}\left(W_{c}z\right)$$

and a scalar TVC regressor

(8) $${\hat{y}}_{\mathrm{TVC}}=w_{r}^{⊤}z$$

The two heads are trained jointly with a class-weighted cross-entropy and a mean-squared TVC term:

(9) $$L={\mathrm{CE}}_{w}\;\left({\hat{y}}_{\mathrm{cls}},y\right)+\lambda \;{\left({\hat{y}}_{\mathrm{TVC}}-\mathrm{TVC}\right)}^{2}$$

Both models use the same 60 min windows and the same folds. Class weighting follows Section 3.3: it is applied to the network, while for the trees, the weighted and unweighted fits are equivalent. Three uses of TVC must be distinguished, since only the first is leakage. TVC as an input feature hands the model the quantity from which the label is derived. TVC as an auxiliary regression target adds supervision without exposing the answer at inference. Classification supervised by class labels alone is the purest comparison. The network above uses TVC in the second sense only, and Section 4.2 reports an ablation in which the regression head is removed entirely. Several front-end and training variants of the deep model were additionally evaluated. These were wavelet denoising, per-window normalisation, $R/R_{0}$ baseline correction, and domain-adversarial training. None improved on the configuration reported here, so the base configuration is used throughout.

For the rich-signal foil, each trace is a 10-step heater profile waveform in which the position of a reading within the profile is discriminative. Two versions of the same backbone are therefore evaluated. The position-aware version adds a learned positional embedding and a two-layer transformer encoder and keeps a position-preserving head. The position-blind version removes the positional embedding and aggregates the sequence with a learned attention pool. This makes the representation invariant to step order. Both are trained identically. Foil models are compared by macro-F1 under stratified five-fold cross-validation repeated over three seeds, with the tree baseline evaluated on the same folds.

### 3.5. Implementation and Settings

All experiments ran on a commodity CPU-only workstation (no GPU). Classical models used the scikit-learn and XGBoost libraries. The deep model used PyTorch (Meta Platforms, Inc., Menlo Park, CA, USA). All were ran using Python (Python Software Foundation, Wilmington, DE, USA). The key windowing, tree, and network settings are listed in Table 2; both models are evaluated on the identical windows and folds of Section 3.3.

### 3.6. Channel Importance, Deployment, and the Dose-Response

Channel importance and sensor selection are nested inside the training folds. In each outer LOCO fold, the leave-one-sensor-out ranking is computed by an inner leave-one-cut-out procedure over the eleven training cuts only. The five highest-ranked channels are chosen from that inner evaluation alone, and the resulting array is scored on the held-out cut, which never participates in the selection. Selection is thus repeated independently twelve times, and the frequency with which each channel is chosen is reported alongside accuracy. For reference, the subset obtained by ranking on all twelve folds at once, as in the original analysis, is also evaluated. Deployment cost is reported as serialised model size and per-window inference latency for both models. The signal-richness criterion is tested while holding chemistry, class count, and sample size fixed. On CoffeePow-4, a single factor is varied: the 10-step heater profile is down-sampled to L∈{10,7,5,3,1} evenly spaced steps. The tree and the position-aware network are re-evaluated at each *L*. If the deep advantage is really about temporal richness, it should be present while the profile is rich. It should vanish at L=1, where no temporal structure remains.

## 4. Results

### 4.1. Under Leakage-Free Validation, Trees Win Significantly

Table 3 reports the matched-fold LOCO comparison with sensor signals as the only inputs. The gradient-boosted-tree pipeline is the stronger model on every aggregate metric. It reaches an accuracy of 0.81±0.10, macro-F1 of 0.68±0.15, balanced accuracy of 0.70, MCC of 0.66, quadratic-weighted κ=0.87, and pooled TVC $R^{2}=0.77$. The deep network reaches a macro-F1 of 0.50±0.15. Because the two models are evaluated on identical folds and windows, the difference can be tested directly. The tree wins on all 12 cuts. The paired Wilcoxon signed-rank test gives p<0.001 for macro-F1 (median paired difference 0.198, 95% CI [0.124,0.251]) and p<0.001 for accuracy; with twelve folds, this is the smallest attainable two-sided value. Other classical models on the same windows and folds behave similarly (random forest: 0.65; LightGBM: 0.65; multilayer perceptron: 0.62; support vector machine: 0.61; logistic regression: 0.59 macro-F1), all above the deep network. Per-class F1 (Table 4) shows that the difficulty is concentrated in the acceptable grade, which overlaps the spoiled grade in TVC. Both models separate the extremes well and confuse the middle, but the tree does so less.

**Figure 4:**
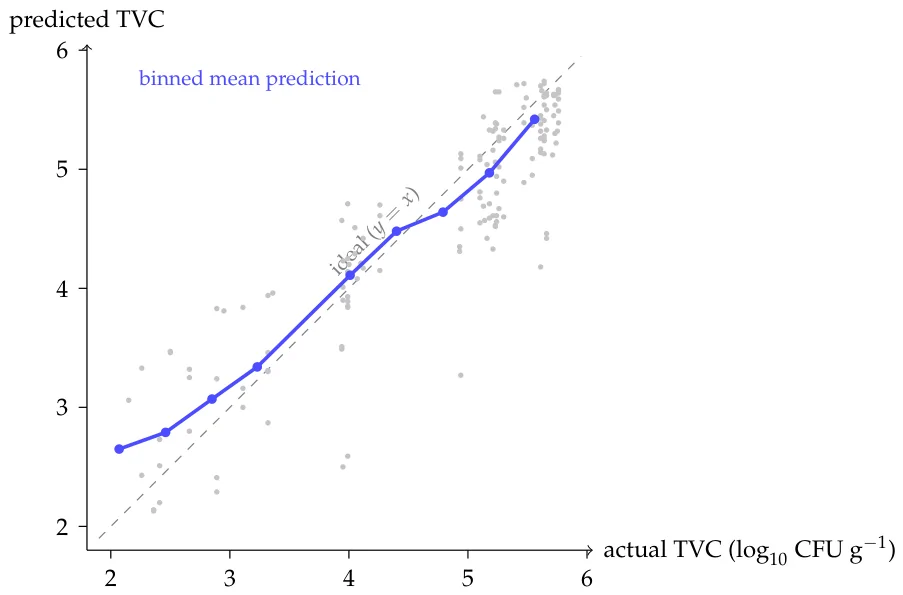
Out-of-fold TVC regression under LOCO (gradient-boosted trees; pooled $R^{2}=0.77$). Grey: 150-window sample; dashed: y=x; blue: mean prediction per actual-TVC bin. Fitted slope 0.80 (regression toward the mean).

The regression head is well calibrated through the middle of the spoilage range, but compresses at the extremes (Figure 4). A line fitted to the pooled out-of-fold predictions has a slope ogf 0.80, so the model regresses toward the mean. It over-predicts TVC for fresh meat by roughly 0.3–$0.5\;\log_{10}$ CFU $g^{-1}$. It under-predicts it for heavily spoiled meat by roughly 0.2–0.3. The under-prediction at high microbial load is the deployment-relevant direction. It argues for a conservative decision threshold when the array is used as a freshness screen. The over-prediction at the fresh end, by contrast, carries a purely economic cost. Section 4.5 makes the resulting operating point quantitative. Flagging sound meat as approaching spoilage produces false positives that translate directly into avoidable food waste. The two error directions therefore warrant asymmetric weighting in any deployed threshold.

### 4.2. Impact of Input Representation and Auxiliary Multi-Task Supervision

The comparison in Table 3 sets a feature-based pipeline against an end-to-end one, so the gap may arise from the feature representation rather than from the model family. Two further arms separate these. The first is a neural network that receives the same 55 window summaries while keeping the deep model’s shared representation, heads, optimiser, and class weighting. The second concatenates those summaries into the shared representation of the multi-scale network. Both use the identical folds, windows, and per-fold seeds. Table 5 reports the outcome. The feature-matched network exceeds the raw-window network by a median 0.175 macro-F1 (p=0.007), whereas the difference between the tree and the feature-matched network is a median 0.021 and is not significant (p=0.204). The greater part of the original gap is therefore attributable to the representation. What survives is a narrower and more defensible claim: end-to-end learning does not recover these summaries from sixty-minute windows sampled once per minute. Feature fusion performs worse than the summaries alone, which is consistent with the convolutional branch supplying cut-specific level information that does not generalise to a held-out cut.

The same table reports an ablation in which the TVC regression head is removed so that class labels alone supervise training. The auxiliary head confers no significant advantage: the paired difference is a median 0.017 macro-F1 with p=0.233, and the multi-task variant wins on 8 out of 12 folds. In this purest comparison, the tree still exceeds the network by a median 0.246 macro-F1 (p<0.001). The auxiliary supervision therefore does not confound the comparison of Section 4.1.

### 4.3. Reproducing the Near-Perfect Numbers: Leakage and Random Splitting

**Figure 5:**
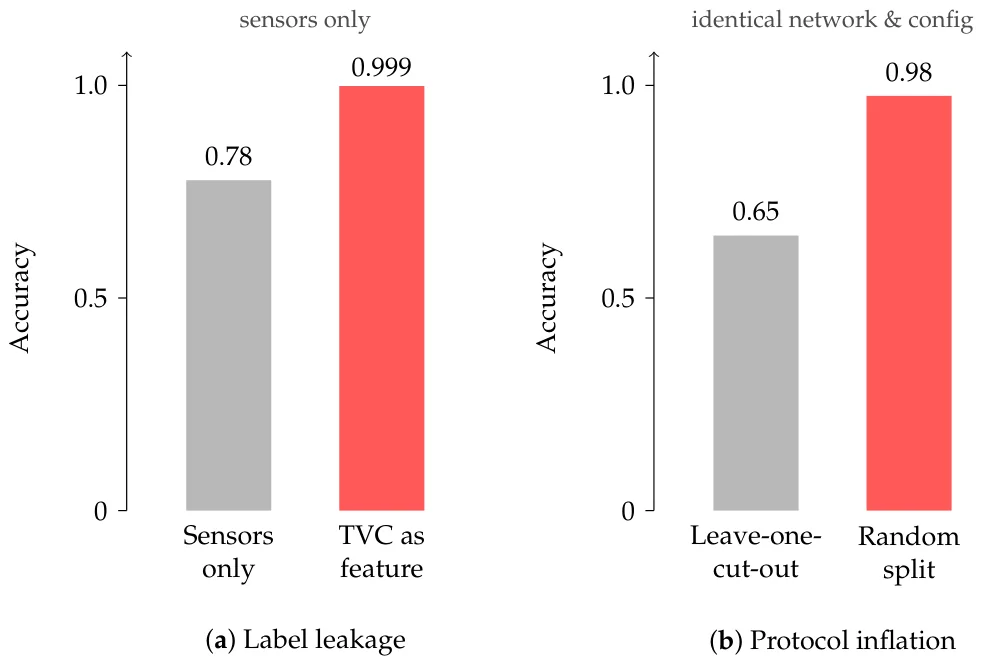
Two single-variable changes that inflate accuracy on the beef data. (**a**) Adding TVC as an input feature lifts a per-timestep random forest from 0.78 to 0.999. (**b**) Switching from leave-one-cut-out to a random split lifts the same network from 0.65 to 0.98.

Figure 5 and Table 6 isolate the two conditions that produce inflated accuracy. First, when TVC is supplied as an input feature, the model is handed the very quantity from which the class is derived. A per-timestep random forest that scores 0.777 on sensor signals alone then reaches an accuracy of 0.9985; the classifier re-reads its own label. Supplying TVC as an input feature is therefore sufficient to produce near-perfect accuracy on this corpus. Whether it accounts for any particular published figure cannot be established without access to the corresponding validation procedures, and no such claim is made here. Second, the network and inputs are held fixed. Only the evaluation is changed from LOCO to a stratified five-fold random split (each fold an 80/20 partition). This lifts the same multi-scale attention network from macro-F1 0.50 (accuracy 0.65) to 0.97±0.01 (accuracy 0.98±0.01). Nothing about the sensor, the model, the hyperparameters, or the biology changed, only the way the data were divided. The 94.83% four-class accuracy of the grouped-evaluation reference model [15] is consistent with this being the realistic upper range for the task. The 99.8–100% figures reported elsewhere [12,13,14] are consistent with the inflated regime, although other factors may also contribute. These include feature selection or hyperparameter tuning carried out on the full dataset, binary rather than four-grade task definitions, and differences in the handling of class imbalance.

### 4.4. Robustness, a Reduced Array, and Deployment Cost

**Figure 6:**
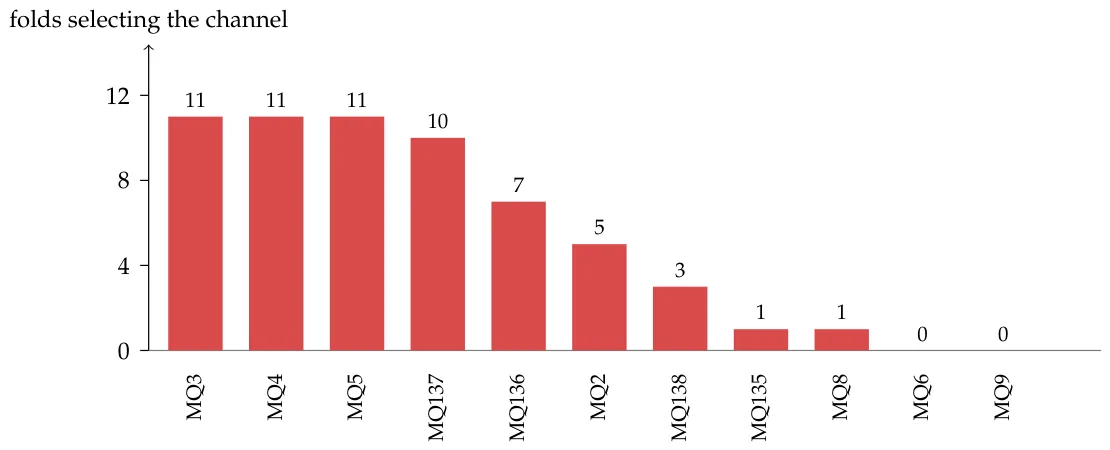
Selection stability under nested sensor selection: the number of the twelve outer folds in which each channel entered the five-sensor subset chosen from the training cuts alone. The ranking is reproducible even though the reduced array does not outperform the full array.

Table 7 reports three practical results. Cross-dataset transfer is difficult. A model trained on the primary corpus and applied to an independent nine-sensor acquisition drops well below its within-corpus score. This is expected when sensor units and calibration differ. The deep network still transfers better (macro-F1 0.46) than the tree (0.40). Its learned temporal features depend less on absolute sensor magnitude than the tree’s level-based window summaries. The size and origin of this gap and the calibration-transfer strategies that close much of it are analysed in Section 5. Sensor selection nested within the training folds gives a more sober picture than the original analysis. The nested five-sensor array reaches macro-F1 0.64±0.17 against 0.68±0.15 for the full array—a difference that is not significant (median −0.022; p=0.240). The subset fixed a priori from the earlier, non-nested ranking reaches 0.70±0.15, which is likewise indistinguishable from the full array (p=0.831). A reduced array therefore costs nothing measurable, but neither does it improve on the full array: the advantage reported previously was an artefact of ranking and evaluating on the same folds. The ranking itself is stable (Figure 6). MQ3, MQ4 and MQ5 are selected in 11 out of 12 folds and MQ137 in 10, while MQ6 and MQ9 are never selected. The chemistry is consistent with this ordering. MQ137 responds to ammonia and volatile amines, the products of the proteolytic route that dominates late storage [3], and MQ3 to alcohols and aldehydes, which accumulate throughout [1]. MQ4 and MQ5 are nominally hydrocarbon detectors and plausibly report light volatiles of lipid oxidation, although their broad cross-sensitivity means part of their contribution may be an indirect response to the same amine and alcohol fraction. MQ136 ranks below these four because hydrogen sulphide is produced mainly by organisms that are not dominant under aerobic chilled storage [1], so sulphur volatiles stay low relative to the amine fraction; it is nevertheless selected in 7 out of 12 folds. For MQ138, the likelier explanation is redundancy, since its target classes overlap those of MQ3. These are interpretations consistent with the ranking, not chemical speciation, which the corpus does not provide. By summary type, the window mean carries 46% of the tree’s gain, and the final value a further 21%. Standard deviation, range, and slope together account for the remaining third. The two strongest individual features are the mean responses of MQ4 and MQ5. The static level dominates the intra-window dynamics, with slope being the least useful summary. This is itself consistent with the coarse-sampling account. A sixty-minute window of once-a-minute readings carries little waveform structure for a slope or range feature to exploit. Finally, both models are edge-feasible. The tree runs in 0.013 ms per window and the deep network in 0.50 ms. The deep network is the smaller on disk (107,418 parameters, 420 kB vs. 1.8 MB), but both are well within microcontroller budgets. Given its higher accuracy and lower latency, the tree remains the preferred choice for on-site deployment on a single calibrated device; Section 5 qualifies this for transfer to a differently calibrated one.

### 4.5. An Operating Point for a Conservative Screen

The under-prediction at high microbial load has a direct operational consequence, which can be stated quantitatively. Table 8 sweeps the decision point of the pooled out-of-fold TVC prediction, taking a positive to be an actual TVC of at least 5.0 $\log_{10}$ CFU $g^{-1}$ (1980 of 3252 windows). At the nominal threshold, the screen misses just over a quarter of truly spoiled windows. Moving the decision point to a predicted TVC between 4.35 and 4.55, a margin of 0.45 to 0.65 $\log_{10}$ CFU $g^{-1}$, raises sensitivity from 0.74 to between 0.92 and 0.96 at a cost of 0.12 to 0.22 in specificity. An equivalent rule on the classifier output triggers when the spoiled-class probability reaches 0.20. The limit of the instrument should be stated with equal clarity: 99% sensitivity is not attainable at any threshold in the sweep, with the maximum being 0.989.

### 4.6. When the Signal Is Rich, the Deep Model Helps: A Signal-Richness Criterion

The beef result raises the question of whether the deep model is simply weak or if the beef signal is unsuited to it. To separate these, the same architecture is applied to a substrate in which each measurement is a ten-step heater profile waveform (Table 9). On the four-class task, the position-aware network reaches macro-F1 0.967±0.009. This is above both the tree (0.935±0.008; paired p<0.001) and its own position-blind variant (0.954±0.009). The originating study reports 0.964 for a compact transformer under a comparable protocol [17], so this value is on par with that reference. The gap between the position-aware and position-blind variants shows that step position carries usable information. The effect is modest, about 1.3 points, not the collapse a naive pooling would suggest. On the harder seven-class task, the tree is again the stronger model (0.960 vs. 0.950). Temporal richness is therefore a precondition for the deep model to win, not a guarantee of it.

**Figure 7:**
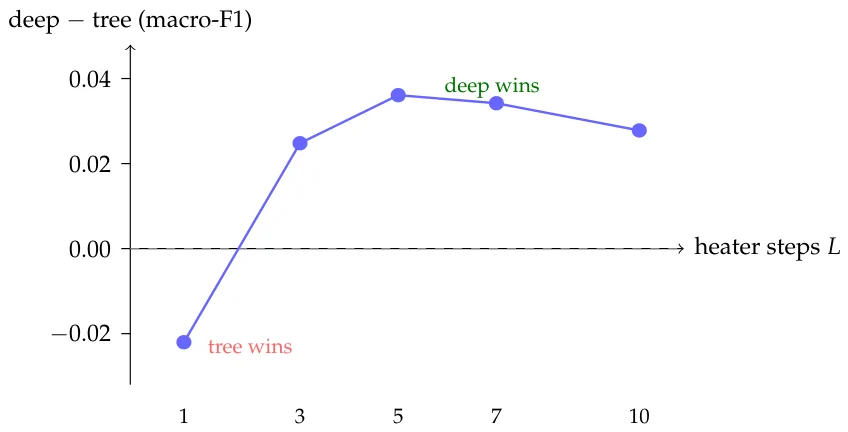
Signal-richness dose-response on CoffeePow-4, with chemistry, classes, and sample sizes fixed. The position-aware network’s advantage over the tree falls as heater steps *L* decrease, and reverses at L=1 (the beef regime).

The test isolates whether the deep advantage is about temporal richness. Chemistry, classes, and sample size are held fixed, and only the number of heater steps retained is varied (Figure 7). The advantage of the position-aware network over the tree is positive, while the profile is rich (+0.036 at five steps). It falls to −0.022 at a single step, where the measurement carries no internal waveform structure and the tree wins. That single-step case is exactly the beef regime. This dose-response supports a simple signal-richness criterion. A deep temporal model can help only when each measurement is a waveform with informative internal structure. It offers nothing over a gradient-boosted tree when the interior of the analysis window carries no such structure, as when each measurement is a single reading. The beef corpus, sampled once per minute, sits at the single-reading end; the BME688 profiles sit at the rich end.

## 5. Discussion

The practical message is that evaluation and representation, rather than architecture, are the decisive variables for MOS-array e-nose meat freshness assessment as currently reported. Once TVC is withheld and cross-validation respects sample boundaries, the accuracy of a low-cost beef e-nose is about 0.81, not 1.00. The strongest model is then a gradient-boosted tree rather than a deep network, by a statistically significant margin. This is consistent with broader evidence that gradient-boosted trees remain a strong baseline for tabular feature vectors [70]. This is not a negative result. An accuracy of 0.81 with quadratic-weighted κ=0.87 and TVC $R^{2}=0.77$ is a usable screening capability for food quality control. It is obtained from an inexpensive array under uncontrolled ambient conditions and validated on entirely unseen cuts. Nested selection shows that a five-sensor array costs nothing measurable relative to the full eleven, although it does not improve on it. A subset ablated in software is, moreover, not a device. Physically removing sensors alters chamber volume, gas flow, thermal load, and the calibration procedure, none of which channel ablation captures. The claim is therefore one about bill of materials, and a physical five-sensor array remains to be built and validated.

The decomposition of Section 4.2 sharpens what this comparison establishes. The tree does not beat the network because trees are inherently better suited to gas sensor arrays. It beats the network because five per-channel summaries capture what a sixty-minute window of once-a-minute readings contains, and a network given those same summaries performs no differently from the tree. The finding is therefore about the representation the measurement admits, not about model families, and it is the same conclusion the signal-richness dose-response reaches from the opposite direction.

One boundary of the application should be stated explicitly. Total viable count measures overall microbial load and, therefore, freshness and remaining shelf life. It is not a proxy for the presence of pathogens or their toxins, and contamination can occur without perceptible spoilage odour or a correspondingly elevated aerobic count. The instrument evaluated here is a quality and shelf-life screen. The conservative operating point of Section 4.5 protects against shipping product past its shelf life; it is not a safety guarantee, and it should not be presented as one.

The errors that remain are concentrated where the biology is genuinely continuous. The hardest grade is acceptable. Its total viable count overlaps that of the spoiled class near the spoilage threshold of about $10^{5}$ CFU $g^{-1}$. This is expected rather than a model artefact. The transition from acceptable to spoiled beef is driven by the same spoilage microbiota and their volatile products. These include biogenic amines, sulphur compounds, and ammonia, accumulating continuously. The odour fingerprint of late-acceptable and early-spoiled meat is therefore chemically contiguous rather than distinct. No gas sensor model should be expected to draw a sharp boundary there. The ordinal metrics reflect this. The quadratic-weighted κ=0.87 is high precisely because the residual confusions fall between adjacent grades, not between fresh and spoiled.

The signal-richness criterion explains an apparent tension. A deep model was effective for tea grading [16], and deep learning successes are now common in food quality control [4,5]. The difference is the signal, not the food. The tea and coffee measurements are heater-profile waveforms whose shape carries the discriminative information. The beef measurements are single readings sampled once per minute along a slow spoilage trajectory. The dose-response makes this concrete. On one dataset, holding everything else fixed, the deep advantage persists while the profile retains several steps. It reverses at a single step, where no structure remains inside the measurement. This also reconciles an apparent counter-example. The denoising long–short-term-memory model of Wijaya et al. [15] is a deep model, uses grouped evaluation, and reports 94.83% four-class accuracy on this corpus. It consumes the full 2220 min trajectory in which the temporal structure of spoilage resides, whereas the windowed protocol adopted here discards that structure by construction. Its result is therefore consistent with the signal-richness criterion rather than being contrary to it. The criterion for practitioners is to match model complexity to the temporal richness of the measurement. A waveform model should not be expected to aid with coarsely-sampled data.

The cross-dataset drop was analysed rather than left as a caveat. Over the nine shared channels, the discrepancy between corpora is large and significant: the squared maximum mean discrepancy (MMD^2^) is 0.55±0.01 (radial basis kernel, median-heuristic bandwidth, permutation p=0.005), and it is concentrated rather than diffuse. Removing MQ5 improves transfer by 0.146 macro-F1 and removing MQ136 by 0.036, whereas removing MQ4 costs 0.157; MQ5 is thus the principal carrier of the gap despite being among the strongest channels within a single corpus. Three calibration-transfer strategies were then evaluated. Per-cut $R/R_{0}$ referencing does not help, and the distribution analysis explains why: after $R/R_{0}$, the discrepancy rises slightly to 0.591. Standardising each device’s channel statistics before inference raises the network from macro-F1 0.464 to 0.633 (accuracy 0.682), and domain-adversarial training [47] reaches 0.616 while reducing the discrepancy in the learned representation from 0.267 to 0.176. The tree gains from neither, consistent with its reliance on absolute response levels. Two qualifications apply. Both effective strategies are transductive, using the unlabelled target recordings, so deployment assumes that a calibration sample can be taken from the new device. Moreover, 0.633 remains below the within-corpus figure. The conclusion is that the gap is substantially a calibration problem rather than a failure of the freshness signal, and that, for transfer to an independently calibrated device, the learned representation with per-device standardisation is the better choice, whereas within one device, the tree is preferable.

Practical deployment raises three further issues. On calibration, the analysis above indicates that between-device differences are largely affine, so a short calibration recording should form part of any deployment procedure. On environmental variability, the primary corpus was acquired under uncontrolled ambient conditions, which aids external validity but prevents temperature and humidity from being treated as controlled factors; the secondary corpus records both, and explicit compensation is a natural next step. On drift, each acquisition spans 37 h, far shorter than the timescale over which metal-oxide baselines drift, so this corpus cannot characterise long-term drift, and no claim about it is made. Periodic recalibration against a fresh-state reference is recommended, with the caveat established here that $R/R_{0}$ referencing alone does not solve the between-device problem. The reduced array should likewise be validated per device rather than assumed to transfer, since MQ5, one of its members, is also the least portable channel.

Several limitations bound these conclusions. The primary corpus is a single meat type acquired by one group. As Section 3 reports, its TVC series is, moreover, shared across ten of the twelve cuts, so the study validates across twelve independent sensor acquisitions, but not across twelve independent microbiological trajectories. Broader validation across laboratories and storage regimes, and over longer horizons where sensor drift becomes significant, is also needed. The rich-signal foil is a different food matrix used only to vary signal richness under controlled conditions. It is not itself a meat-quality target. Finally, the signal-richness criterion is supported by one controlled dose-response and two substrates. A fuller test across several intermediate-richness e-nose datasets is a natural next step.

## 6. Conclusions

Beef freshness assessment was benchmarked with a low-cost MOS gas sensor array under a leakage-free, group-structured protocol. A gradient-boosted-tree pipeline predicted freshness class (accuracy 0.81±0.10, macro-F1 0.68±0.15, quadratic-weighted κ=0.87), and microbial load ($R^{2}=0.77$). It significantly outperformed a multi-scale attention convolutional network (paired Wilcoxon p<0.001). A network supplied with the same window summaries was statistically indistinguishable from the tree, which locates the advantage in the feature representation rather than in the model family. Using the same data, the field’s near-perfect accuracies were reproduced by two single-variable changes. The first supplied TVC as a feature (0.999). The second replaced grouped validation with a random split, the same network rising from 0.50 to 0.97 macro-F1. This shows that those figures reflect the evaluation, not the device. Under nested, per-fold selection, a five-sensor subset was statistically indistinguishable from the full array, so fewer than half the channels suffice at no measurable cost, although a physical reduced array remains to be built and validated.For deployment as a conservative screen, the decision point should sit at a predicted TVC of 4.35–4.55, a margin of 0.45–0.65 $\log_{10}$ CFU $g^{-1}$ below the nominal threshold, which raises sensitivity from 0.74 to between 0.92 and 0.96. Finally, a controlled dose-response was run on a rich BME688 heater profile dataset. A position-aware network exceeds the tree, and the advantage disappears as the profile is shortened to a single step. This established a signal-richness criterion for when a deep temporal model is warranted. That criterion concerns the measurement rather than the meat. It is therefore expected to extend to other matrices, such as poultry and pork read by similar MOS arrays. At the same coarse sampling, a compact model should suffice. Justifying a deep temporal model would require a richer acquisition, such as heater profile modulation. This prediction remains to be tested across matrices. The contribution is a defensible, edge-deployable pipeline for meat freshness monitoring, together with the evaluation methodology and model selection criterion that reliable food-quality deployment requires.

## Figures and Tables

**Table 1:** Sensor array: nominal target analytes and the classes of spoilage volatiles each channel reports.

Channel	Nominal Target	Spoilage Volatiles Reported
MQ137	Ammonia	Ammonia, volatile amines (cadaverine, putrescine)
MQ136	Hydrogen sulfide	Sulfur volatiles
MQ138	Aromatics, ketones, alcohols	Alcohols, ketones, aromatic volatiles
MQ3	Alcohols	Alcohols, short-chain aldehydes
MQ135	Air quality (${\mathrm{NO}}_{x}$, ${\mathrm{CO}}_{2}$, benzene, NH_3_)	Mixed; secondary ammonia response
MQ2	Combustible gases, LPG	Short-chain hydrocarbons
MQ4	Methane, natural gas	Short-chain hydrocarbons of lipid oxidation
MQ5	LPG, natural gas	Short-chain hydrocarbons
MQ6	LPG, butane	Short-chain hydrocarbons
MQ8	Hydrogen	Hydrogen, fermentative products
MQ9	Carbon monoxide, methane	Carbon monoxide, short-chain hydrocarbons

**Table 2:** Windowing, model, and training settings.

Setting	Value
Software	Python 3.12.3; scikit-learn 1.2.2; XGBoost 3.2.0; PyTorch 2.6.0 (CPU); LightGBM 4.6.0; SciPy 1.17.1
Window length/stride	60 min/8 min
Tree features (per sensor)	mean, standard deviation, final value, range, slope
Gradient-boosted trees	400 estimators, max depth 6, learning rate 0.08, subsample 0.9
MS-CNN-Attention stem	branch kernels {3,7,15}, branch width b=48 ($C^{\prime }=144$)
SE reduction/attention	reduction ratio 4; single-head temporal self-attention
Optimiser	Adam, learning rate $1.5\times 10^{-3}$ (cosine-annealed), weight decay $10^{-4}$
Batch size/epochs	128/25
Multi-task weight λ	0.5 (TVC term); class-weighted cross-entropy
Foil validation	stratified 5-fold × 3 seeds (macro-F1)

**Table 3:** Matched-fold LOCO comparison on the beef data (12 folds), sensor signals only. Last column: paired Wilcoxon test across folds.

Metric	GBT (Window Summary)	MS-CNN-Attention	Paired *p*
Accuracy	0.81 ± 0.10	0.65 ± 0.18	<0.001
Macro-F1	0.68 ± 0.15	0.50 ± 0.15	<0.001
Balanced accuracy	0.70	0.59	—
MCC	0.66	0.45	—
Quadratic-weighted κ	0.87	0.72	—
TVC RMSE ($\log_{10}$ CFU $g^{-1}$)	0.48	0.62	—
TVC $R^{2}$ (pooled)	0.77	0.60	—

**Table 4:** Per-class F1 under LOCO (pooled out-of-fold predictions).

Model	Excellent	Good	Acceptable	Spoiled	Macro
GBT (window summary)	0.75	0.67	0.49	0.91	0.71
MS-CNN-Attention	0.65	0.51	0.30	0.78	0.56
MS-CNN-Attention (no TVC head)	0.57	0.44	0.29	0.76	0.51

**Table 5:** Representation versus architecture and the effect of the auxiliary TVC head. All arms use identical folds, windows, and per-fold seeds.

Model	Macro-F1	Accuracy	TVC $R^{2}$
GBT, 55 window summaries	0.68 ± 0.15	0.81 ± 0.10	0.77
Neural network, same 55 summaries	0.64 ± 0.17	0.76 ± 0.12	0.71
MS-CNN-Attention with feature fusion	0.54 ± 0.17	0.68 ± 0.17	0.68
MS-CNN-Attention, raw windows	0.50 ± 0.15	0.65 ± 0.18	0.60
MS-CNN-Attention, no TVC head	0.46 ± 0.15	0.61 ± 0.17	—

Paired Wilcoxon: feature-matched network versus raw windows p=0.007; GBT versus feature-matched network p=0.204 (not significant).With versus without the TVC head p=0.233 (not significant).

**Table 6:** Controlled reproduction of inflated performance; each row changes one thing relative to Table 3.

Change	Model	Accuracy	Macro-F1
None (leakage-free LOCO)	MS-CNN-Attention	0.65	0.50
Random split instead of LOCO	MS-CNN-Attention	0.98	0.97
None (leakage-free LOCO), per-timestep	Random forest	0.78	0.63
TVC supplied as an input feature, per-timestep	Random forest	0.999	0.997

**Table 7:** Robustness and deployment. Transfer: trained on the primary corpus, tested on the independent 9-sensor corpus over shared channels.

Quantity	GBT (Window Summary)	MS-CNN-Attention
Cross-dataset transfer, macro-F1	0.40	0.46
Nested 5-sensor array, LOCO macro-F1	0.64	—
Full 11-sensor array, LOCO macro-F1	0.68	0.50
Inference latency (ms/window)	0.013	0.50
Model size	1.8 MB	420 kB

**Table 8:** Operating points for a conservative freshness screen. Positive = actual TVC ≥5.0$\log_{10}$ CFU $g^{-1}$; pooled out-of-fold predictions under LOCO (n=3252, 1980 positive).

Decision Rule	Sensitivity	Specificity	PPV	False Negatives
Predicted TVC ≥5.00 (nominal)	0.738	0.924	0.938	519
Predicted TVC ≥4.55 (maximum Youden)	0.921	0.800	0.877	156
Predicted TVC ≥4.35	0.958	0.708	0.836	83
Spoiled-class probability ≥0.20	0.953	0.778	0.870	94

**Table 9:** Rich-signal foil: BME688 heater profile classification (macro-F1, five-fold × three seeds). ConvTran: value reported by the dataset’s originating study.

Model	CoffeePow-4	Aroma-7
GBT (tabular)	0.935 ± 0.008	0.960 ± 0.005
MS-CNN-Attention (position-blind)	0.954 ± 0.009	0.910 ± 0.012
MS-CNN-Attention (position-aware)	0.967 ± 0.009	0.950 ± 0.008
ConvTran (reference [17])	0.964	—

## Data Availability

The primary beef data were obtained from the work of Wijaya [66] and are freely available at https://dataverse.harvard.edu/dataset.xhtml?persistentId=doi:10.7910/DVN/XNFVTS (accessed on 7 July 2026). The cross-dataset corpus was obtained from the work of Wijaya et al. [65] and is freely available at https://data.mendeley.com/datasets/mwmhh766fc/3 (accessed on 7 July 2026). The BME688 data were obtained from the work of Stefanone et al. [17] and are freely available at https://zenodo.org/records/15425922 (accessed on 7 July 2026). All source code, the exact dataset identifiers and fold definitions, the model settings and random seeds, the per-window out-of-fold prediction files, and a pinned software environment (Python 3.12.3; XGBoost 3.2.0, scikit-learn 1.2.2, PyTorch 2.6.0, LightGBM 4.6.0, NumPy 1.26.4, SciPy 1.17.1, pandas 2.3.3) are freely available at https://github.com/eco160/beef-enose-leakage-free (accessed on 7 July 2026) and permanently archived on Zenodo (https://doi.org/10.5281/zenodo.21718733), enabling full reproduction of every table and figure.

## Abbreviations

The following abbreviations are used in this manuscript:

MOS	Metal-oxide semiconductor
e-nose	Electronic nose
TVC	Total viable count
LOCO	Leave-one-cut-out (cross-validation)
CNN	Convolutional neural network
GBT	Gradient-boosted trees
SE	Squeeze-and-excitation
MCC	Matthews correlation coefficient
VOC	Volatile organic compound
MMD	Maximum mean discrepancy
